# Exploring the complex role of the Eph/Ephrin signaling in oral and maxillofacial cancers

**DOI:** 10.3389/fonc.2025.1554751

**Published:** 2025-05-12

**Authors:** Reydson Alcides de Lima-Souza, Moisés Willian Aparecido Gonçalves, Raisa Sales de Sá, Luccas Lavareze, João Figueira Scarini, Talita de Carvalho Kimura, Fernanda Cristina Poscai Ribeiro, Albina Altemani, Fernanda Viviane Mariano, Gary Chris Fillmore, Erika Said Abu Egal

**Affiliations:** ^1^ Department of Pathology, School of Medical Sciences, University of Campinas (FCM/UNICAMP), Campinas, São Paulo, Brazil; ^2^ Department of Oral Diagnosis, Piracicaba School of Dentistry, University of Campinas (FOP/UNICAMP), Piracicaba, São Paulo, Brazil; ^3^ Department of Internal Medicine, University of Western São Paulo (UNOESTE), Guarujá, São Paulo, Brazil; ^4^ Biorepository and Molecular Pathology, Huntsman Cancer Institute, University of Utah (UU), Salt Lake City, UT, United States

**Keywords:** Eph, ephrin, oral squamous cell carcinoma, salivary gland cancers, target therapy

## Abstract

The Eph (erythropoietin-producing hepatocellular carcinoma) receptor family represents the largest subgroup within the tyrosine kinase receptor family and is recognized for its critical role in regulating the growth, migration, and survival of both normal and malignant cells. The Eph/ephrin signaling has an ambiguous role in squamous cell carcinomas of the oral region, playing both a suppressive and oncogenic role. In salivary gland cancers, the results are reserved, although they suggest that some molecules are associated with a worse prognosis for patients. This review offers a comprehensive summary of the existing literature, underscoring the evidence that supports the involvement of the Eph/ephrin signaling in oral and maxillofacial cancers. Additionally, we examine molecular discoveries that may present promising therapeutic targets for these malignancies.

## Graphical Abstract

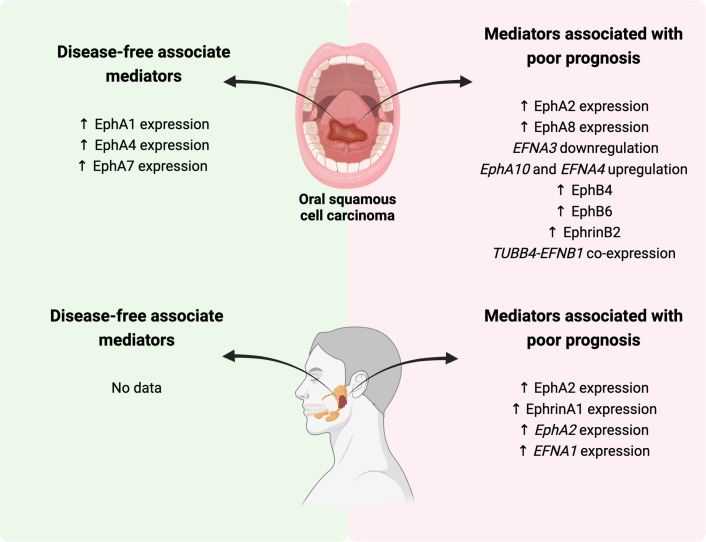

## 1 Introduction

Oral and maxillofacial (OMF) cancers are a growing number of neoplasms, mainly affecting the oral cavity, lips, oropharynx, salivary glands, and maxillary bones. Among the subtypes of cancers in this region, squamous cell carcinoma (SCC) is the main histological subtype, affecting mainly men over the fifth decade of life, smokers, and alcoholics (1). In addition to SCC, salivary gland cancers (SGCs) are another rare and important subgroup of tumors affecting the OMF region (1). Diagnosing SGCs is challenging due to the diverse subtypes and overlapping morphological features (2).

The Eph (erythropoietin-producing hepatocellular carcinoma) receptor family represents the largest subgroup within the tyrosine kinase receptor family and is recognized for its critical role in regulating the growth, migration, and survival of both normal and malignant cells. Eph receptors act by binding to their ligand, the ephrins (3). The role of the Eph/ephrin signaling has been demonstrated in different types of cancer. In colorectal carcinoma, decreased expression of EphA1 and EphA5 was found to be related to invasion, metastasis, and poor survival (3, 4). Furthermore, in esophageal squamous cell carcinoma, the overexpression of EphA2 was correlated with the disease’s advanced stage (5). EphB4 also contributes to tumor biology, being involved with increased proliferation, motility, and migration of cancer cells (6). Gastric tumors have been shown to correlate EphA3 expression with tumor progression (7, 8).

The Eph/Ephrin signaling system plays a critical role in various physiological and pathological processes, including tumor progression, metastasis, and resistance to therapy (8). While Eph/Ephrin signaling has been studied in various cancers, important gaps remain in our understanding of the precise molecular mechanisms driving these processes, particularly in specific tumor types such as SGCs.

Furthermore, while significant progress has been made in targeting the Eph/Ephrin signaling for therapeutic purposes, challenges such as drug specificity and off-target effects remain largely underexplored. There is also a lack of detailed discussion on how Eph/Ephrin-targeting agents may offer personalized treatment strategies, particularly for oral squamous cell carcinoma (OSCC) and SGC.

This review aims to bridge these knowledge gaps by providing a comprehensive analysis of Eph/Ephrin signaling in OMF cancer biology. By synthesizing recent findings and integrating molecular, clinical, and therapeutic perspectives, we provide new insights into the complex role of Eph/Ephrin in tumor progression and its potential as a therapeutic target.

## 2 Materials and methods

Electronic searches were conducted in the databases used for reference sourcing, including PubMed, Scopus, and Web of Science, without restrictions on language or publication date. The search keywords, such as “Eph receptors,” “ephrin signaling,” “oral cancer,” “head and neck squamous cell carcinoma,” and “salivary gland cancer” were used, combined with boolean operators (OR, AND). Furthermore, the references of the included articles were manually reviewed to identify potential additional studies. Articles focusing on the Eph/ephrin system concerning oral SCC and SGC were included.

## 3 Overview

### 3.1 The role of EPH receptors and ephrin in cancer

Erythropoietin-producing human hepatocellular (Eph) receptors constitute a significant family of receptor tyrosine kinases, classified into EphA and EphB subclasses (8). The EphA subclass includes nine receptors (EphA1–8 and EphA10), while the EphB subclass comprises five members (EphB1–4 and EphB6) (9). These receptors contain extracellular domains that detect environmental signals, thereby influencing cell interactions and migration. The Eph/ephrin signaling enables bidirectional signaling, where both Eph receptors and ephrins can act as receptors or ligands (10). In forward signaling, which primarily occurs through phosphoserine-dependent pathways, Eph receptor activation triggers intracellular cascades involving molecules such as Janus kinase (JAK)/signal transducer and activator of transcription (STAT), Rho and Ras family GTPases, focal adhesion kinase (FAK), and phosphoinositide 3-kinase (PI3K). In contrast, reverse signaling occurs in the ephrin-expressing cell, initiated by the phosphorylation of tyrosine residues in the cytoplasmic tail of B-ephrins, activating downstream signaling effectors and intracellular cascades (10). Notably, Eph receptors can also function independently of kinase activity, being modulated by other receptor tyrosine kinases (RTKs) (11).

Eph receptor ligands are classified into two types: Ephrin-A ligands, which are anchored to the cell surface by glycosylphosphatidylinositol (GPI), and Ephrin-B ligands, which possess a transmembrane domain and a brief cytoplasmic segment (8, 12). Eph receptors interact with ephrins, which are categorized into two classes: ephrin-A (ephrin-A1 to A6) and ephrin-B (ephrin-B1 to B3) (13). Typically, A-type receptors can bind to a majority or all A-type ligands, whereas B-type receptors are usually able to bind to a majority or all B-type ligands (14).

Eph receptors and their ligands, ephrins, participate in a dynamic system involving both ligand-dependent and ligand-independent signaling. In ligand-dependent signaling, Eph receptors bind to ephrins on neighboring cells, triggering receptor clustering and phosphorylation, which activate downstream pathways. In contrast, ligand-independent signaling occurs without ephrin interaction, where Eph receptors can function autonomously. Notably, in cancer cells with low Eph receptor phosphorylation, tumor-promoting effects are likely driven by mechanisms that do not require ephrin stimulation (15).

Since their discovery, Eph receptor tyrosine kinases have been linked to various physiological processes (16). They play a critical role in cell migration and adhesion, which are essential for cellular organization during development. Activation of Eph receptors or ephrins can induce either cell repulsion or adhesion and invasion (16).

Despite their crucial roles in normal physiology, the involvement of Eph family members in cancer is complex and often contradictory. Research suggests that Eph/ephrin bidirectional signaling affects cell communication, regulating migration, adhesion, differentiation, and proliferation (17). For example, EphrinA1 is present at vasculogenesis sites in embryos and on tumor cells in various cancers, including breast cancer (18). Furthermore, EphA2 has been shown to inhibit many angiogenic functions of vascular endothelial growth factor (VEGF) (19).

Additionally, EphA2 stimulation decreases FAK phosphorylation, thereby inhibiting integrin-mediated cell adhesion (20). As critical transmembrane receptors, integrins regulate cellular responses to the tumor microenvironment and facilitate intercellular communication across various cell types (21). Conversely, activation of EphA2 by ephrinA1 can attenuate Ras activation, suppress the Akt–mTORC1 pathway, and inhibit cell migration (22). In summary, the contradictory roles of Eph receptors and ephrins in cancer may result from the diversity of Eph signaling pathways and the heterogeneity of cancer microenvironments (17).

## 4 Eph receptors and ephrin in oral and maxillofacial cancers

### 4.1 Oral squamous cell carcinoma

OSCC is considered a major global health problem, according to Globocan, there are an estimated 350,000 new cases of oral cavity cancer and 170,000 deaths worldwide, 77% of which occur in developing countries. OSCC progression typically begins with epithelial cell hyperplasia, followed by dysplasia, carcinoma *in situ*, and eventually invasive carcinoma. Well-differentiated tumors mimic stratified epithelium, showing mature cells in layered arrangements with keratin pearls, while poorly differentiated tumors contain immature cells with nuclear pleomorphism and atypical mitoses, lacking organized layers and keratinization (23).

With this variance, it is essential to study new therapeutic approaches and prognostic markers (24). Ephs/ephrin signaling was initially shown to participate across a broad spectrum in the developmental process, being able to regulate cell adhesion, migration or chemo-repulsion, and tissue/cell boundary formation (25, 26). The role of Eph receptors and their ephrin ligands in oral cancer provide diverse approaches to understanding the principal associations to tumor growth, invasion, metastasis, and angiogenesis (**Table 1**). Moreover, unlike traditional oncogenes that often function only in tumor cells, Eph receptors mediate cell-to-cell interactions in both tumor cells and the tumor microenvironment and are considered attractive targets for drug design (26).

**Table 1:** Eph expression and role in oral squamous cell carcinoma.

Eph receptors	Study Method	Association with OSCC	Reference
EphA1	IHC; 37 samples	High expression of EphA1 was significantly linked to the absence of vascular invasion and lymph node metastases.	(26)
EphA2	IHC; 59 samples	EphA2 expression was correlated with shorter survival periods and was a significant prognostic factor in oral cancer.	(27)
EphA4	IHC; 37 samples	Elevated EphA4 expression was significantly associated with the absence of lymph node metastases.	(26)
EphA7	IHC; 37 samples	EphA7 was a favorable prognostic factor for overall and disease-free survival.	(26)
EphA8	IHC, *in vitro* assay; 119 samples	EphA8 overexpression potentially accelerates OSCC progression by augmenting tumor cell invasion, rather than proliferation capacity.	(31)
EphrinA4	IHC; *In vitro* assay; *In vivo* model	EphrinA4 has been linked to poor recurrence-free survival in patients with OSCCs.	(33)
EphB4	IHC; 73 samples	EphB4 showed a significant correlation with tumor recurrence.	(29)
EphB6	IHC; 54 samples	High EphB6 expression was significantly associated with advanced tumor staging and lymph node metastasis, as well as poorer patient outcomes, leading to higher mortality rates.	(34)
EphrinB2	IHC; 73 samples	EphrinB2 showed a significant correlation with depth of invasion.	(29)
*EFNA3*	IHC; *In vitro* assay; *In vivo* model; 53 samples	*EFNA3* is crucial in the progression of OSCC, indicating ephrinA3 as a promising target for oral cancer treatment.	(32)
*EFNB1*	IHC; *In vitro* assay; *In vivo* model; 12 samples	*EFNB1* is correlated with a poorer prognosis.	(36)

IHC, Immunohistochemistry; OSCC, oral squamous cell carcinoma.

#### 4.1.1 Ephrin-A and EphA

**Figure 1:**
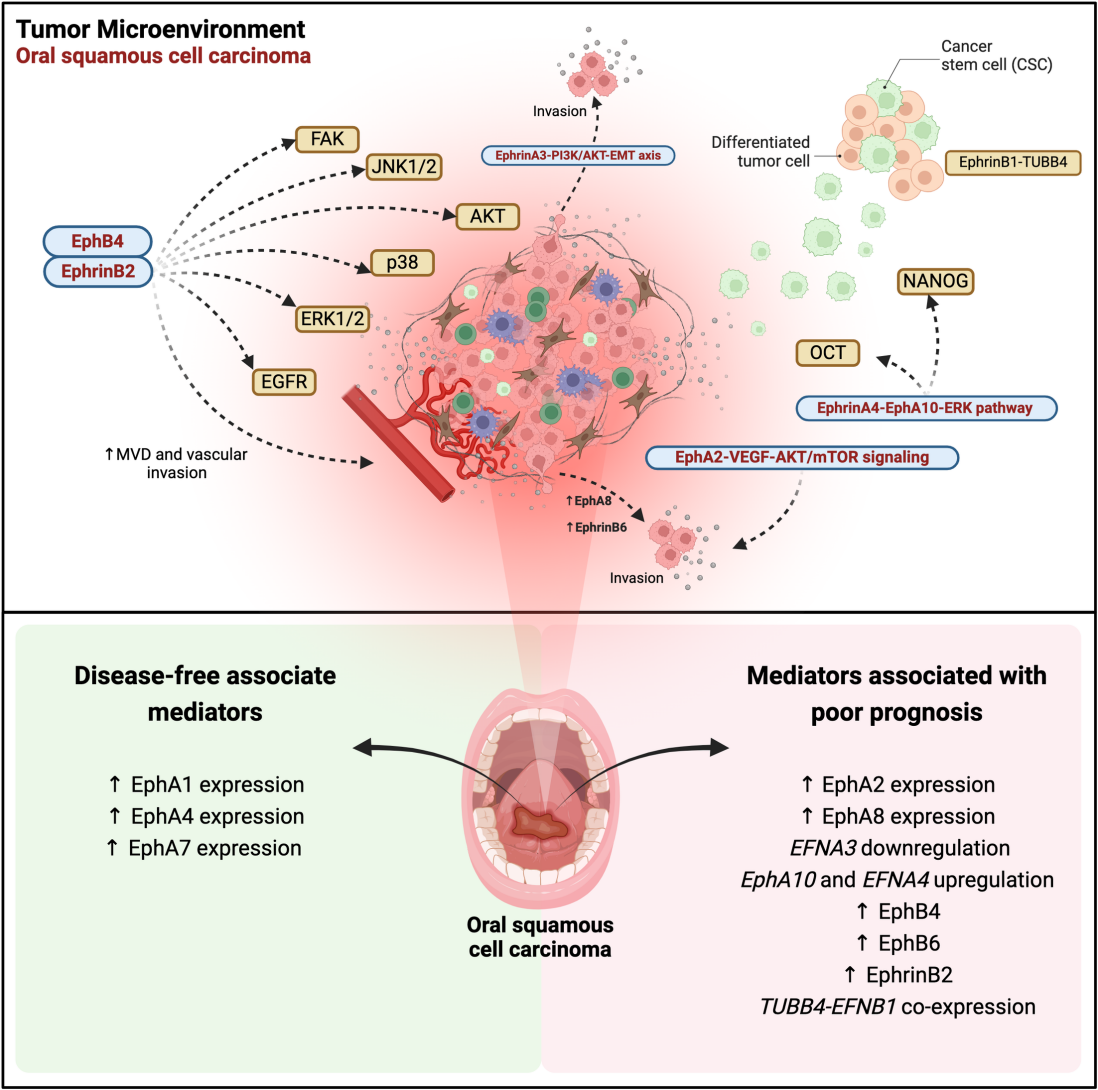
Eph/ephrin signaling in oral squamous cell carcinoma (OSCC): regulatory pathways, tumor progression, and therapeutic implications. EphA2-VEGF-AKT/mTOR signaling promotes proliferation, migration, invasion, and apoptosis inhibition. The EphrinA3-PI3K/AKT-EMT axis drives epithelial-mesenchymal transition (EMT) and tumor invasion. EphrinA4-EphA10 interaction activates ERK, inducing cancer stem cell (CSC) expansion via OCT4 and NANOG. EphA8 overexpression enhances tumor invasion. The EphB4-ephrinB2 pathway regulates microvascular density (MVD) and vascular invasion, activating multiple pro-migratory pathways, including EGFR, FAK, ERK1/2, p38, AKT, and JNK1/2. EphB6 overexpression is associated with tumor progression and metastasis, while the EphrinB1-TUBB4B complex sustains the CSC niche and worsens prognosis. In the lower panel, Eph receptors and ephrin ligands associated with disease-free survival are highlighted, along with mediators correlated with prognosis in OSCC. The data reflects gene expression and immunohistochemistry levels reported in various studies, illustrating their correlation with clinical outcomes in affected patients. Created in BioRender. Egal, E. (2025) https://BioRender.com/f96q435.

Shao et al. investigated the Eph/Ephrin interaction in OSCC through the immunoexpression of EphA2 and VEGF in 59 cases of tongue cancer and 10 cases of normal oral mucosa. The results demonstrated that elevated levels of EphA2 and VEGF, as well as increased microvessel density (MVD) and advanced TNM (tumor size, nodal spread, metastasis) stage, were associated with shorter survival periods and served as significant negative prognostic factors in oral cancer (**Figure 1**). These findings underscore the potential clinical benefits of combination therapy targeting both EphA2 and VEGF signaling pathways (27).

A recent *in vitro* study using Cal-27 cells confirmed the overexpression of EphA2 and EphA4 in OSCC. The data showed that the activation of the AKT/mTOR signaling pathway was increased, leading to the suppression of cell cycle arrest and apoptosis. Additionally, when AKT/mTOR inhibitors were used to block this pathway, the effect of overexpression was reversed. Therefore, EphA2 promotes the proliferation, migration, and invasion of Cal-27 cells and inhibits apoptosis by enhancing the AKT/mTOR signaling pathway (28).

A broader study evaluating the EphA immunoexpression of EphA1, A2, A4, and A7 in 37 tongue SCC specimens, in association with clinicopathological parameters as well as with overall survival and disease-free survival revealed that EphA7 was a favorable prognostic factor for overall and disease-free survival (26) (**Figure 1**). On the other hand, the expression of EphA1, A2, and A4 showed no significant association with overall and disease-free survival. High expression of EphA1 was significantly linked to the absence of vascular invasion and lymph node metastases (**Figure 1**). Similarly, elevated EphA4 expression was significantly associated with the absence of lymph node metastases (**Figure 1**). The elevated expression of EphA2 was significantly associated with a dense stromal inflammatory reaction (26). Corroborating with these data, Saito et al, evaluated EphA2 expression it showed to be associated with the malignant potential of the oral epithelium (29).

When evaluated in laryngeal SCC, *EPHA7* was found to be upregulated *in vitro*. Therefore, the authors found that the downregulation of *EPHA7* inhibits cell growth and proliferation by promoting apoptosis, emerging as a therapeutic potential for human LSCC (30).

With regards to gene and protein expression of EphA8, it was found to be highly expressed in OSCC tissues. In addition, EphA8 was considered to be an independent prognostic factor in the advanced stage of this tumor (31) (**Figure 1**). *In vitro* experiments corroborated these findings, demonstrating that EphA8 overexpression potentially accelerates OSCC progression by augmenting tumor cell invasion, rather than proliferation capacity when using SCC-25 and H357 cancer cells (31).

The role of ephrinA3 in tumor occurrence and progression is not well established. *EFNA3* together with *HOXA1, HOXA9, HOXA3*, and *E3F3* are part of the miR-210-3p gene, and *EFNA3* is regulated by this miRNA under hypoxic conditions. Also, this ligand plays a significant role in regulating the biological behavior of oral cancer cells, particularly through its involvement in epithelial-mesenchymal transition (EMT) via the PI3K/AKT signaling pathway. MiR-210-3p directly targets the *EFNA3* gene, leading to decreased expression of ephrinA3 expression. This downregulation, facilitated by the upregulation of miR-210-3p, influences the biological behavior of these cancer cells. Thus, the miR-210-3p-EphrinA3-PI3K/AKT signaling axis is crucial in the progression of OSCC, indicating ephrinA3 as a promising target for oral cancer treatment (32) (**Figure 1**).

Cancer stem-like cells (CSCs) have emerged as key players in metastasis and tumor recurrence, contributing significantly to the high morbidity associated with oral cancer. Transcription factors such as Octamer-binding 4 (OCT4) and NANOG play crucial roles in CSC maintenance. Recent studies have highlighted the involvement of the ephrin A4-EphA10 axis in mediating direct signaling, which regulates cell migration, and sphere formation, and positively regulates NANOG and OCT4 transcription factors, via ERK activation. Moreover, the high co-expression of ephrinA4 with NANOG or OCT4 has been linked to poor recurrence-free survival in patients with OSCCs. Interestingly, inhibiting this interaction through ectopic expression of EphA10 suggests that the upregulation of *EPHA10* and *EFNA4* in OSCC tissues enhances cis interactions. Additionally, the upregulation of *EFNA4* in cancer tissues alone appears sufficient to induce downstream effects of direct ephrinA4-EphA10 signaling (33) (**Figure 1**).

#### 4.1.2 Ephrin-B and EphB

One of the first studies to evaluate OSCC was that by Dong et al. analyzing the immunoexpression of EphB6 in 54 samples of this tumor. This study demonstrated that high EphB6 expression was significantly associated with advanced tumor staging and lymph node metastasis, as well as poorer patient outcomes, leading to higher mortality rates (**Figure 1**). The results showed that EphB6 can be used as a new prognostic marker for this tumor (34).

Recently, the expression of EphA2, EphB4, and ephrinB2 in 73 patients with OSCC and 43 patients with potentially malignant oral disorders (POMD) was studied by IHC. Positivity for these markers was found in epithelial cells and some stromal vascular cells, in proportion to the level of malignancy. In addition, EphrinB2 was significantly higher in patients without recurrence than in those with recurrence. Furthermore, high EphB4 expression was associated with depth of invasion (**Figure 1**). In summary, immunoexpression of EphB4, EphB2, MVD, and lymphatic vessel density (LVD) was associated with the malignant potential of the oral epithelium (29).

Another important study showed that overexpression of ephrinB2 in OSCC cells is related to tumor progression, lymph node metastasis, and unfavorable survival outcomes. Moreover, increased ephrinB2 levels were observed in OSCC cell lines compared to normal human oral keratinocytes, correlating with the migratory and invasive potential of OSCC cell lines. Transfection of an *EFNB2*-specific small interfering RNA (siRNA) into SAS-L1 cells resulted in a significant reduction in proliferation, adhesion, migration, and invasion, achieved by inhibiting phosphorylation of epidermal growth factor receptors (EGFR), FAK, and the signaling pathways ERK1/2, p38, AKT, and JNK1/2. Additionally, *EFNB2* suppression notably reduced the adhesion and transmigration of SAS-L1 cells towards human lymphatic endothelial cells. In summary, these findings suggest that ephrinB2 overexpression and activation of the *EFNB2* signaling pathway in the TME of OSCC promote lymph node metastasis and progression, thereby enhancing malignant potential and interaction with adjacent cells (35) (**Figure 1**).

Dharmapal et al. investigated the co-expression of TUBB4 (Tubulin Beta 4B Class IVb), an isotype of β-tubulin that is related to the maintenance of cell morphology, and ephrinB1 in the membranes of CSCs in oral cancer. *In vitro* immunofluorescence analysis revealed that TUBB4 and ephrinB1 co-localize within the CSC niche, forming a gradient that supports CSC maintenance. Additionally, reverse immunoprecipitation of ephrinB1 confirmed its dependency on TUBB4B expression. Thus, TUBB4B regulates the membrane expression of ephrinB1, thereby influencing CSC signaling. Furthermore, these results indicated that the cooperation between *TUBB4B* and *EFNB1* is correlated with a poorer prognosis (36) (**Figure 1**).

Preclinical and clinical studies have investigated the use of EphB4-Human Serum Albumin Fusion Protein (sEphB4-HAS) in the treatment of cancer, particularly head and neck squamous cell carcinoma (HNSCC). The study by Bhatia et al. (2016) demonstrated that sEphB4-HAS effectively inhibits tumor growth and enhances radiosensitivity in HNSCC xenograft models, showing promising results both as monotherapy and in combination with radiotherapy (37). Bhatia et al. (2019) reinforced these findings, observing that the combination of sEphB4-HAS with radiotherapy potentiated the therapeutic response, leading to a significant reduction in tumor growth (38). It has been established that sEphB4-HAS, when combined with other treatments like chemotherapy and radiation, exhibited synergistic effects, increasing apoptosis and inhibiting tumor cell proliferation (39). Finally, the clinical study by El-Khoueiry et al. (2016), which investigated the safety and efficacy of sEphB4-HAS in patients with advanced solid tumors in head and neck, showed that the therapy was well-tolerated and, in two of the 17 patients, stable disease control was observed, suggesting that this approach has clinical potential (40). These studies indicate that targeting the EphB4 receptor may be a promising strategy for the treatment of HNSCC and other solid tumors.

Although EphB4-targeting agents show positive effects in reducing tumor growth, especially in combination with radiotherapy, drug specificity remains a significant challenge. This is because Eph receptors are widely expressed in various tissues and normal cells, which may lead to off-target effects and cause unexpected side effects, such as vascular or neurological alterations. Therefore, more clinical studies are needed to confirm their efficacy and safety, as well as to assess long-term impacts in a larger and more heterogeneous patient population (37, 40).

### 4.2 Salivary gland cancers

SGCs are a heterogeneous group of tumors, with differing biological behaviors and treatment responses, comprising around 5% of head and neck tumors (41). According to the World Health Organization (WHO), the most common malignant tumors of the salivary glands are mucoepidermoid carcinoma and adenoid cystic carcinoma (AdCC) (1).

Regarding SGCs, the Eph-Ephirn studies are only focused on AdCC. This tumor is characterized by its slow, invasive, and progressive growth associated with local recurrences and distant metastases (42, 43). AdCC is composed of epithelial and myoepithelial cells that arrange themselves in a cribriform, tubular, and solid pattern, with the solid pattern most often associated with a worse prognosis (42, 43).

Given that EphA2 and ephrinA1 play an important role in tumor angiogenesis, Shao and co-workers (2013), studied the association of EphA2 and ephrinA1 in AdCC. The results evidenced high protein and mRNA expression of frozen AdCC tissues when compared with frozen normal salivary gland tissues. Immunohistochemical analysis revealed intense staining in the tumor cells when compared to normal tissue and nerves in cases where the neural invasion was present (44).

**Figure 2:**
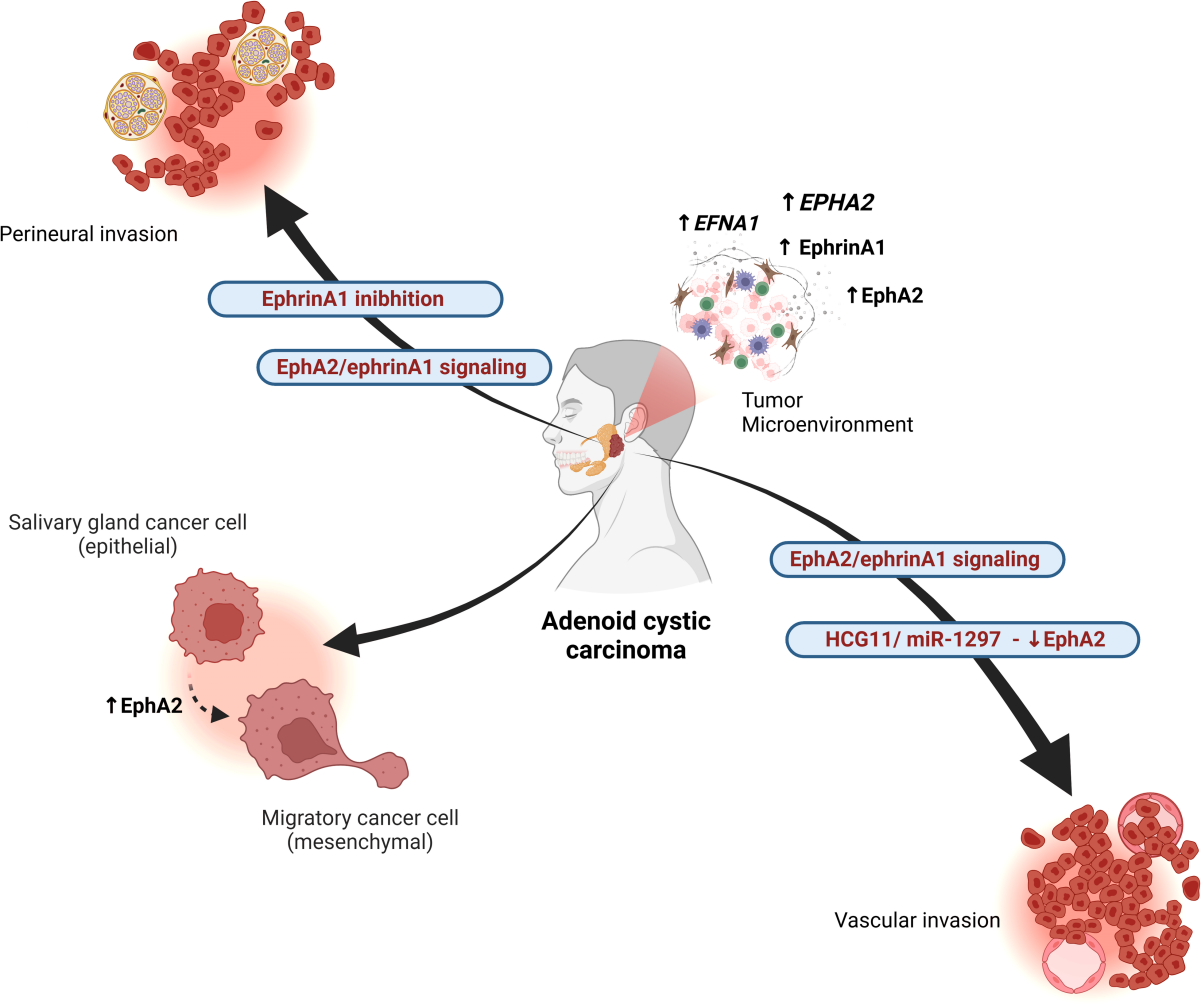
Interactions of the Eph/ephrin system in adenoid cystic carcinoma (AdCC). The EphA2/ephrinA1 signaling pathway regulates tumor angiogenesis and is associated with advanced TNM stage, perineural invasion, and vascular invasion. EphA2 expression is increased in the solid pattern of basaloid AdCC, correlating with greater aggressiveness. EphA2 overexpression reduces cell proliferation, migration, and invasion in AdCC. The long non-coding RNA HCG11 regulates miR-1297, which negatively controls EphA2, contributing to tumor progression. Additionally, EphA2 modulates epithelial and mesenchymal markers, promoting epithelial-mesenchymal transition (EMT) in AdCC. The absence of ephrinA1 is associated with perineural spread. Overall, the Eph/ephrin axis influences tumor heterogeneity by regulating the expression of various family members, including *EFNA1*, *EPHA2*, EphrinA1, and EphA2. Created in BioRender. Egal, E. (2025) https://BioRender.com/f96q435.

After checking the expression of EphA and ephrinA1 in AdCC samples, the association of EphA2, ephrinA1, and MVD expression with the clinicopathological features of AdCC was analyzed. Among the 3 histological patterns of AdCC, the expression of EphA2, ephrinA1, and MVD was significantly higher in the solid pattern than in the tubular and cribriform pattern. Furthermore, EphA2 and ephrinA1 expression and MVD were found to correlate with TNM clinical stage, perineural invasion, and vascular invasion. Such results suggest that EphA2 and ephrinA1 contribute to AdCC progression by promoting AdCC angiogenesis and may serve as therapeutic targets for this tumor (44) (**Figure 2**).

Yan and Wang (2023) evaluated the expression levels of the long non-coding RNA HCG11, microRNA-1297 (miR-1297), and EphA2 in AdCC cell lines compared to normal human salivary gland (HSG) cell lines using quantitative reverse transcription PCR. EphA2 protein levels were assessed via western blotting. The results showed that overexpression of EphA2 inhibited the proliferation, migration, and invasion of AdCC cells. Their findings suggest that the HCG11/miR-1297/EphA2 regulatory axis in AdCC could be a potential target for new therapeutic approaches to treat this condition (45).

Interestingly, Fukai et al. (2014), reported a case of AdCC with perineural dissemination through the mandibular nerve to the region of the middle cranial fossa and right infratemporal fossa. Aiming at further understanding of the case, epithelial and mesenchymal markers were evaluated. Immunoreactivity of transcription factors involved in the EMT was evidenced. The presence and absence of EphA2 and ephrinA1 expression in AdCC tumor cells respectively was observed by immunohistochemical analysis. *EPHA2* was identified in frozen tissue by real-time reverse transcriptase polymerase chain reaction, with higher mRNA expression in tumor tissue than in normal salivary glands (46).

In summary, studies on Eph-Ephrin in AdCC, revealed heightened expression of EphA2 and ephrinA1, particularly in the solid pattern, correlating with tumor aggressiveness. Elevated levels of EphA2 and ephrinA1 were associated with advanced clinicopathological features like TNM stage, perineural invasion, and vascular invasion, indicating their involvement in promoting AdCC angiogenesis. Additionally, EphA2 expression was linked to perineural dissemination in AdCC, suggesting its potential as a therapeutic target in managing this tumor (**Figure 2**). Despite a thorough literature review, no studies specifically addressing the role of the Eph/Ephrin system in other SGC were found. This highlights a significant gap in the current understanding of Eph/Ephrin signaling in SGCs, reinforcing the need for future research in this area.

## 5 Eph/Ephrin signaling and cancer ecosystem

Studies have suggested that cancer should be viewed as a multidimensional pathological ecosystem, where ecological interactions and evolutionary processes play crucial roles in tumor progression, therapy resistance, and disease recurrence (47). This perspective emphasizes the importance of considering not only somatic mutations and molecular mechanisms but also the spatial and temporal dynamics of the tumor and its microenvironment (47).

The Eph/ephrin system, known for regulating cell-cell interactions, cell migration, invasion, and angiogenesis, exemplifies how this ecological and evolutionary approach applies to cancer (8, 9). Studies indicate that Ephrin A4-EPHA10 signaling is associated with OSCC progression, impacting recurrence-free survival in patients with high *EFNA4* co-expression with NANOG or OCT4 (33). Additionally, inhibition of the EphB4-ephrin-B2 pathway in experimental HNSCC models led to the reprogramming of the tumor immune microenvironment, suggesting that targeting this system could alter tumor ecology and reduce immune evasion (38). In SGC, EphA2 and ephrinA1 markers have been associated with MVD, indicating a critical role in tumor angiogenesis. These findings reinforce the hypothesis that Eph/ephrin signaling contributes to tumor progression by modulating the microenvironment, making it a potentially relevant therapeutic target (44).

By adopting the perspective that cancer is a pathological ecosystem, we can better understand how Eph/ephrin-mediated interactions contribute to tumor heterogeneity, therapeutic resistance, and the evolutionary adaptation of oral and maxillofacial cancers. This integrated approach may pave the way for more effective therapeutic strategies that consider not only molecular targets but also the complex ecological and evolutionary interactions within the tumor (47).

## 6 Conclusion and future directions

The role of the Eph/Ephrin signaling in the development and progression of oral and maxillofacial cancers is ambiguous. In oral SCCs, while EphA2, EphB4, and ephrinB2 are associated with a worse prognosis, EphA1, EphA4, and EphA7 play the opposite role, being related to a more favorable prognosis. For SGC, the results are reserved but suggest an important role for EphA2 and ephrinA1 in contributing to AdCC progression by promoting angiogenesis.

The Eph/ephrin system is particularly compelling as a therapeutic target because of its dual function in regulating tumor-promoting and tumor-suppressing signals. This makes it an attractive candidate for precision medicine, where targeting specific Eph/ephrin interactions could provide more effective and less toxic treatment options for patients with OMF cancers.

Future research on Eph/ephrin-targeting therapies should focus on identifying biomarkers for patient stratification, optimizing drug selectivity and efficacy, and exploring combination therapies to enhance outcomes. Addressing drug resistance and conducting larger, biomarker-driven clinical trials will also be essential to confirm the safety and long-term benefits of these therapies. These efforts will guide the development of more personalized and effective cancer treatments.

## References

[1] WHO. WHO Classification of Tumours Editorial Board. Head and neck tumours. 5th ed. WHO classification of tumours series. Lyon (France): International Agency for Research on Cancer (2023).

[2] Stenman G Persson F Andersson MK. Diagnostic and therapeutic implications of new molecular biomarkers in salivary gland cancers. Oncol. (2014) 50:683–90. doi: 10.1016/j.oraloncology.2014.04.008 24856188

[3] Dong Y Wang J Sheng Z Li G Ma H Wang X. Downregulation of EphA1 in colorectal carcinomas correlates with invasion and metastasis. Modern Pathol. (2009) 22:151–60. doi: 10.1038/modpathol.2008.188 19011600

[4] Gu S Feng J Jin Q Wang W Zhang S. Reduced expression of EphA5 is associated with lymph node metastasis, advanced TNM stage, and poor prognosis in colorectal carcinoma. Histol Histopathol. (2017) 32:491–7. doi: 10.14670/HH-11-815 27651378

[5] Miyazaki T Kato H Kimura H Inose T Faried A Sohda M. Evaluation of tumor Malignancy in esophageal squamous cell carcinoma using different characteristic factors. Anticancer Res. (2005) 25:4005–11. 16309192

[6] Tachibana M Tonomoto Y Hyakudomi R Hyakudomi M Hattori S Ueda S. Expression and prognostic significance of EFNB2 and EphB4 genes in patients with oesophageal squamous cell carcinoma. Digest Liver Dis. (2007) 39:725–32. doi: 10.1016/j.dld.2007.05.013 17611172

[7] Lv X Wang J Huang F Wang P Zhou J Wei B. EphA3 contributes to tumor growth and angiogenesis in human gastric cancer cells. Oncol Rep. (2018). 2408–2416. doi: 10.3892/or.2018.6586 30066881

[8] Buckens OJ El Hassouni B Giovannetti E Peters GJ. The role of Eph receptors in cancer and how to target them: novel approaches in cancer treatment. Expert Opin Invest Drugs. (2020) 29:567–82. doi: 10.1080/13543784.2020.1762566 32348169

[9] Gucciardo E Sugiyama N Lehti K. Eph- and ephrin-dependent mechanisms in tumor and stem cell dynamics. Cell Mol Life Sci. (2014) 71:3685–710. doi: 10.1007/s00018-014-1633-0 PMC11113620 24794629

[10] Surawska H Ma PC Salgia R. The role of ephrins and Eph receptors in cancer. Cytok Growth Factor Rev. (2004) 15:419–33. doi: 10.1016/j.cytogfr.2004.09.002 15561600

[11] Brantley-Sieders DM Zhuang G Hicks D Bin FW Hwang Y Cates JMM. The receptor tyrosine kinase EphA2 promotes mammary adenocarcinoma tumorigenesis and metastatic progression in mice by amplifying ErbB2 signaling. J Clin Invest. (2008) 118:64–78. doi: 10.1172/JCI33154 18079969 PMC2129239

[12] Tang FHF Davis D Arap W Pasqualini R Staquicini FI. Eph receptors as cancer targets for antibody-based therapy. Advances in Cancer Research (2020), 303–17. doi: 10.1016/bs.acr.2020.04.007 32593404

[13] Arvanitis D Davy A. Eph/ephrin signaling: networks. Genes Dev. (2008) 22:416–29. doi: 10.1101/gad.1630408 PMC2731651 18281458

[14] Papadakos SP Petrogiannopoulos L Pergaris A Theocharis S. The EPH/ephrin system in colorectal cancer. Int J Mol Sci. (2022) 23:2761. doi: 10.3390/ijms23052761 35269901 PMC8910949

[15] Pasquale EB. Eph-ephrin bidirectional signaling in physiology and disease. Cell. (2008) 133:38–52. doi: 10.1016/j.cell.2008.03.011 18394988

[16] Poliakov A Cotrina M Wilkinson DG. Diverse roles of eph receptors and ephrins in the regulation of cell migration and tissue assembly. Dev Cell. (2004) 7:465–80. doi: 10.1016/j.devcel.2004.09.006 15469835

[17] Guo X Yang Y Tang J Xiang J. Ephs in cancer progression: complexity and context-dependent nature in signaling, angiogenesis and immunity. Cell Commun Signaling. (2024) 22:299. doi: 10.1186/s12964-024-01580-3 PMC11137953 38811954

[18] Ogawa K Pasqualini R Lindberg RA Kain R Freeman AL Pasquale EB. The ephrin-A1 ligand and its receptor, EphA2, are expressed during tumor neovascularization. Oncogene. (2000) 19:6043–52. doi: 10.1038/sj.onc.1204004 11146556

[19] Brantley DM Cheng N Thompson EJ Lin Q Brekken RA Thorpe PE. Soluble Eph A receptors inhibit tumor angiogenesis and progression. vivo Oncog. (2002) 21:7011–26. doi: 10.1038/sj.onc.1205679 12370823

[20] Miao H Burnett E Kinch M Simon E Wang B. Activation of EphA2 kinase suppresses integrin function and causes focal-adhesion-kinase dephosphorylation. Nat Cell Biol. (2000) 2:62–9. doi: 10.1038/35000008 10655584

[21] Longmate W DiPersio CM. Beyond adhesion: emerging roles for integrins in control of the tumor microenvironment. F1000Res. (2017) 6:1612. doi: 10.12688/f1000research.11877.1 29026524 PMC5583736

[22] Chen Z Oh D Biswas KH Yu C-H Zaidel-Bar R Groves JT. Spatially modulated ephrinA1:EphA2 signaling increases local contractility and global focal adhesion dynamics to promote cell motility. Proc Natl Acad Sci. (2018) 115:E5696–E5705. doi: 10.1073/pnas.1719961115 PMC6016825 29866846

[23] Johnson DE Burtness B Leemans CR Lui VWY Bauman JE Grandis JR. Head and neck squamous cell carcinoma. Nat Rev Dis Primers. (2020) 6:92. doi: 10.1038/s41572-020-00224-3 33243986 PMC7944998

[24] Bray F Ferlay J Soerjomataram I Siegel RL Torre LA Jemal A. Global cancer statistics 2018: GLOBOCAN estimates of incidence and mortality worldwide for 36 cancers in 185 countries. CA Cancer J Clin. (2018) 68:394–424. doi: 10.3322/caac.21492 30207593

[25] Pasquale EB. Eph receptors and ephrins in cancer: bidirectional signalling and beyond. Nat Rev Cancer. (2010) 10:165–80. doi: 10.1038/nrc2806 PMC2921274 20179713

[26] Theocharis S Klijanienko J Giaginis C Alexandrou P Patsouris E Sastre-Garau X. Ephrin receptor (Eph) -A1, -A2, -A4 and -A7 expression in mobile tongue squamous cell carcinoma: associations with clinicopathological parameters and patients survival. Pathol Oncol Res. (2014) 20:277–84. doi: 10.1007/s12253-013-9692-3 24022400

[27] Shao Z Zhang W-F Chen X-M Shang Z-J. Expression of EphA2 and VEGF in squamous cell carcinoma of the tongue: Correlation with the angiogenesis and clinical outcome. Oncol. (2008) 44:1110–7. doi: 10.1016/j.oraloncology.2008.01.018 18485799

[28] Wang F Zhang H Cheng Z. EPHA2 promotes the invasion and migration of human tongue squamous cell carcinoma cal-27 cells by enhancing AKT/mTOR signaling pathway. BioMed Res Int. (2021) 2021:1–13. doi: 10.1155/2021/4219690 33834064 PMC8016562

[29] Saito H Oikawa M Kouketsu A Takahashi T Kumamoto H. Immunohistochemical assessment of Eph/ephrin expression in oral squamous cell carcinoma and precursor lesions. Odontology. (2020) 108:166–73. doi: 10.1007/s10266-019-00466-y 31654153

[30] Xiang C Lv Y Wei Y Wei J Miao S Mao X. Effect of ephA7 silencing on proliferation, invasion and apoptosis in human laryngeal cancer cell lines hep-2 and AMC-HN-8. Cell Physiol Biochem. (2015) 36:435–45. doi: 10.1159/000430110 25968442

[31] Liu L Wang X Ge W. EphA8 is a prognostic factor for oral tongue squamous cell carcinoma. Med Sci Monitor. (2018) 24:7213–22. doi: 10.12659/MSM.910909 PMC6192305 30300334

[32] Wang L Song Y Wang H Liu K Shao Z Shang Z. MiR-210-3p-EphrinA3-PI3K/AKT axis regulates the progression of oral cancer. J Cell Mol Med. (2020) 24:4011–22. doi: 10.1111/jcmm.15036 PMC7171305 32180353

[33] Chen Y-L Yen Y-C Jang C-W Wang S-H Huang H-T Chen C-H. Ephrin A4-ephrin receptor A10 signaling promotes cell migration and spheroid formation by upregulating NANOG expression in oral squamous cell carcinoma cells. Sci Rep. (2021) 11:644. doi: 10.1038/s41598-020-80060-3 33436772 PMC7804096

[34] Dong Y Pan J Ni Y Huang X Chen X Wang J. High expression of EphB6 protein in tongue squamous cell carcinoma is associated with a poor outcome. Int J Clin Exp Pathol. (2015) 8:11428–33. PMC4637686 26617870

[35] Sasabe E Tomomura A Tomita R Sento S Kitamura N Yamamoto T. Ephrin-B2 reverse signaling regulates progression and lymph node metastasis of oral squamous cell carcinoma. PloS One. (2017) 12:e0188965. doi: 10.1371/journal.pone.0188965 29190834 PMC5708812

[36] Dharmapal D Jyothy A Mohan A Balagopal PG George NA Sebastian P. β-tubulin isotype, TUBB4B, regulates the maintenance of cancer stem cells. Front Oncol. (2021) 11:788024. doi: 10.3389/fonc.2021.788024 35004310 PMC8733585

[37] Bhatia S Hirsch K Sharma J Oweida A Griego A Keysar S. Enhancing radiosensitization in EphB4 receptor-expressing Head and Neck Squamous Cell Carcinomas. Sci Rep. (2016) 6:38792. doi: 10.1038/srep38792 27941840 PMC5150255

[38] Bhatia S Oweida A Lennon S Darragh LB Milner D Phan AV. Inhibition of ephB4–ephrin-B2 signaling reprograms the tumor immune microenvironment in head and neck cancers. Cancer Res. (2019) 79:2722–35. doi: 10.1158/0008-5472.CAN-18-3257 PMC6522285 30894369

[39] Bhatia S Sharma J Bukkapatnam S Oweida A Lennon S Phan A. Inhibition of ephB4–ephrin-B2 signaling enhances response to cetuximab–radiation therapy in head and neck cancers. Clin Cancer Res. (2018) 24:4539–50. doi: 10.1158/1078-0432.CCR-18-0327 PMC6886390 29848571

[40] El-Khoueiry A Gitlitz B Cole S Tsao-Wei D Goldkorn A Quinn D. A first-in-human phase I study of sEphB4-HSA in patients with advanced solid tumors with expansion at the maximum tolerated dose (MTD) or recommended phase II dose (RP2D). Eur J Cancer. (2016) 69:S11. doi: 10.1016/S0959-8049(16)32623-5

[41] Mckenzie J Lockyer J Singh T Nguyen E. Salivary gland tumours: an epidemiological review of non-neoplastic and neoplastic pathology. Br J Maxillofacial Surg. (2023) 61:12–8. doi: 10.1016/j.bjoms.2022.11.281 36623970

[42] da Cruz Perez DE de Abreu Alves F Nobuko Nishimoto I de Almeida OP Kowalski LP. Prognostic factors in head and neck adenoid cystic carcinoma. Oncol. (2006) 42:139–46. doi: 10.1016/j.oraloncology.2005.06.024 16249115

[43] El-Naggar AK Chan JKC Grandis JR Takata T Slootweg PJ. WHO Classification Head Neck Tumours. 4th ed. Lyon: IARC Press (2017).

[44] Shao Z Zhu F Song K Zhang H Liu K Shang Z. EphA2/ephrinA1 mRNA expression and protein production in adenoid cystic carcinoma of salivary gland. J Maxillofacial Surg. (2013) 71:869–78. doi: 10.1016/j.joms.2012.10.026 23298804

[45] Yan S Wang M. HCG11 inhibits salivary adenoid cystic carcinoma by upregulating EphA2 via binding to miR-1297. Surg Med Pathol Radiol. (2023) 135:257–67. doi: 10.1016/j.oooo.2022.08.016 36396591

[46] Fukai J Fujita K Yamoto T Sasaki T Uematsu Y Nakao N. Intracranial extension of adenoid cystic carcinoma: potential involvement of EphA2 expression and epithelial-mesenchymal transition in tumor metastasis: a case report. BMC Res Notes. (2014) 7:131. doi: 10.1186/1756-0500-7-131 24606764 PMC3975335

[47] Luo W. Nasopharyngeal carcinoma ecology theory: cancer as multidimensional spatiotemporal “unity of ecology and evolution” pathological ecosystem. Theranostics. (2023) 13:1607–31. doi: 10.7150/thno.82690 PMC10086202 37056571

